# Patients choose certainty over burden in bladder cancer surveillance

**DOI:** 10.1007/s00345-019-02728-4

**Published:** 2019-03-23

**Authors:** Frits H. M. van Osch, Duncan Nekeman, Neil K. Aaronson, Lucinda J. Billingham, Nicholas D. James, K. K. Cheng, Richard T. Bryan, Maurice P. Zeegers

**Affiliations:** 1 Institute of Cancer and Genomic Sciences, University of Birmingham, Birmingham, UK; 2 Department of Complex Genetics, Nutrition and Translational Research in Metabolism (School NUTRIM), Maastricht University, PO Box 616, 6200 MD Maastricht, The Netherlands; 3 Department of Public Health, Epidemiology and Biostatistics, School of Health and Population Sciences, University of Birmingham, Birmingham, UK; 4 Division of Psychosocial Research and Epidemiology, The Netherlands Cancer Institute, Amsterdam, The Netherlands; 5 Cancer Research UK Clinical Trials Unit, University of Birmingham, Birmingham, UK; 6 Department of Complex Genetics, Nutrition and Metabolism in Translational Research (NUTRIM), Care and Public Health Research Institute (CAPHRI), Maastricht University, Maastricht, The Netherlands

**Keywords:** Non-muscle-invasive bladder cancer, Non-invasive biomarkers, Sensitivity and specificity, Standard gamble

## Abstract

**Background:**

Due to the high risk of recurrence of non-muscle invasive bladder cancer, all patients undergo regular cystoscopic surveillance for early detection. As cystoscopy is invasive, costly and increases the burden of the disease considerably, there is significant ongoing research and development into non-invasive urinary biomarker substitutes. This study aims to assess the level of sensitivity required before patients accept a new urinary biomarker.

**Methods:**

We studied the preferences for a hypothetical diagnostic urinary biomarker and compared this to usual care (cystoscopy) at different levels of sensitivity among 437 patients with bladder cancer (354 men and 83 women) from the UK Bladder Cancer Prognosis Programme. A standard gamble approach was used to estimate the minimally acceptable sensitivity (MAS) of the new biomarker. Additionally, non-parametric statistical analyses were performed to investigate the association between surveillance preference and various patient characteristics.

**Results:**

Almost half of patients (183, 43%) would not replace cystoscopy with a urinary biomarker unless it was 100% sensitive. The median MAS was 99.9999%, and nearly 85% of patients demanded a sensitivity of at least 99% before preferring a urinary biomarker test over cystoscopy. These results were consistent across all patient characteristics and demographic categories.

**Conclusions:**

Our results indicate that patients demand urinary biomarkers as sensitive as cystoscopy before they would be willing to forego cystoscopy for bladder cancer surveillance.

## Introduction

Bladder cancer is the ninth most common malignancy worldwide, with a rising global incidence [1]. The majority of patients (75–80%) present with non-muscle-invasive disease (NMIBC) [2, 3]. Although not immediately life threatening in the majority of cases, recurrence and progression of NMIBC remain significant issues [4, 5], with up to 55% of patients experiencing recurrence within 5 years of diagnosis [6]. Current guidelines recommend long-term surveillance except for low-risk NMIBC after 12 months [5]. With a global prevalence that can be estimated at over 2,000,000, at any one time, there will be a very considerable number of patients requiring such surveillance [1].

Surveillance typically comprises outpatient flexible cystoscopy and urine cytology [5, 7]. For patients with low-risk NMIBC, European Association of Urology (EAU) guidelines recommend follow-up cystoscopy and urine cytology at 3 months and 12 months after tumour resection (TURBT), and then annually for the next 5 years. Patients with high-risk NMIBC undergo more intensive surveillance—every 3 months for the first 2 years, every 6 months until 5 years, and yearly thereafter, most likely for the rest of their lives [5]. If recurrence is detected, then the tumour is resected and surveillance will re-commence from the beginning. It has been estimated that each cystoscopy and urine cytology episode costs £533 [8]; consequently, bladder cancer is the most expensive cancer to treat on a per patient basis from diagnosis to death, with the majority of expense attributable to NMIBC [9, 10].

Cystoscopy itself significantly increases the burden of disease—it is an invasive procedure that causes pain and discomfort in about one-third of patients [11]. In contrast, previous studies have shown that the only burden attributable to a non-invasive test (such as a urinary biomarker) is the waiting time for the test result [11]. For these reasons, a number of diagnostic urinary biomarkers have been developed by academia and industry in an attempt to create less burdensome and less costly NMIBC surveillance regimens.

Urine cytology is widely used as an adjunct to cystoscopy. It has high sensitivity for detecting high-grade disease ( ± 80%), but poor sensitivity for low-grade disease ( ± 30%) [12, 13]; in addition, diagnostic accuracy depends upon a number of confounding factors, such as the quality of the sample and the level of expertise of the cytopathologist [14].

More recently, other types of diagnostic urinary biomarkers have been developed, including soluble urine markers and exfoliated cell markers, as well as multigene urinary DNA-based tests [15, 16]; some are commercially available and US FDA approved [17]. As summarised by Soria et al. in 2018, such biomarkers have moderate to good sensitivity, and multigene panels can reach sensitivity levels of over 90% in patients with high-grade tumours [17]. Biomarker-driven surveillance might become a realistic possibility if, or when, they reach a sufficient and consistent level of sensitivity [18], bearing in mind that cystoscopy itself is operator dependent with the sensitivity and specificity of conventional white light cystoscopy estimated to be up to 85% and 87%, respectively [9, 19].

However, what is less clear is what level of sensitivity is acceptable to patients undergoing surveillance for NMIBC, such that they would be willing to switch from cystoscopy, which in practitioners’ eyes is perceived as the golden standard. Only two studies have previously published on this subject. Vriesema et al., in a utility analysis on 85 patients (70 men and 15 women) undergoing bladder cancer surveillance in The Netherlands, found that 68% of their patients had a minimally acceptable sensitivity (MAS) of over 99% [20]. The more cystoscopies a patient had undergone, the higher was their MAS, suggesting that the acceptability of cystoscopy increases with patients’ familiarity with and/or confidence in the procedure. In a similar utility analysis on 200 patients (119 men and 81 women), Yossepowitch et al. reported that 70% of 200 patients recruited in the United States had a MAS over 99%, and only 24.5% would accept a MAS lower than 95% [21].

The objective of this study was to assess the level of biomarker acceptability in a NMIBC population naïve to conventional cystoscopic surveillance, as well as assessing factors that may affect levels of acceptability, such as socio-demographics and social support.

## Materials and methods

This study is part of the West Midlands Bladder Cancer Prognosis Programme BCPP), which is an ongoing multicentre patient cohort study in the West Midlands region of the UK [22]. Adult patients (age > 18 years) presenting with symptoms suspicious for bladder cancer haematuria in over 80% [23] in the 10 participating urology centres within the region were enrolled on the basis of cystoscopic findings suggestive of bladder cancer. Those who had a previous diagnosis of cancer of the urethra, bladder, ureter or renal pelvis within the last decade, HIV infection, or any other condition that might interfere with the safety of the participant were excluded. The study received ethical approval as part of BCPP (reference: 06/MRE04/65), and written informed consent was obtained from all participants.

At the time of diagnosis, trained research nurses conducted semi-structured face-to-face interviews to collect information on socio-demographics, health-related lifestyle (lifetime smoking history, passive smoking, use of hair dye), medical and drug history, dietary intake, social support and HRQoL. HRQoL was assessed with the European Organisation for Research and Treatment of Cancer EORTC) Quality of Life Questionnaire, the QLQ-C30 [24]. Social support was assessed with the Duke-UNC Functional Social Support Questionnaire FSSQ) [25]. The FSSQ is an eight-item instrument, scored on a 1–5 scale, which measures the strength of a person's social support network. Both the QLQ-C30 and the FSSQ were converted to a scale ranging from 0 to 100, with 0 as the lowest quality of life or lowest level of perceived social support and 100 as the highest. Only the overall HRQoL scale from the QLQ-C30 was used, as we hypothesised that the burden of the cystoscopy would affect overall HRQoL rather than any specific HRQOL domain or symptom. Participants were followed up at approximately 3 months post-baseline measurement with a similar questionnaire. During this follow-up, changes in health-related lifestyle and HRQoL were assessed.

We employed a utility-based patient preference questionnaire to assess the patient’s preference for cystoscopy versus a hypothetical urinary biomarker test. Using a ‘standard gamble’ procedure, we posed a series of questions in which the patient was asked to choose between two surveillance tests, a cystoscopy or a hypothetical urinary biomarker test [26]. The sensitivity of the hypothetical urinary biomarkers started at 100% and decreased in each subsequent question. The definition of sensitivity was explained to the patient as the number of tumours missed by the biomarker out of a thousand. The MAS was defined as the lowest value of accuracy at which the biomarker was favoured over the cystoscopy.

Due to the non-normal distribution of the MAS data, we used non-parametric statistical analyses to investigate the association between surveillance preference and various patient characteristics. This included the Mann–Whitney/Wilcoxon rank-sum test and Spearman's rank correlation coefficient. In addition, we conducted exploratory logistic regression analyses in which we dichotomised the outcome variable (MAS) to identify differences between patients that were willing to sacrifice any sensitivity ( < 100%) and those that were not (100% only). Stratified analyses were used to identify any possible effect modification. Lastly, sensitivity analyses were conducted where patients with an MAS of “0%” and an MAS of “100%” were excluded. All analyses were performed with Stata/MP version 12. A *p* value of < 0.05 was considered statistically significant.

## Results

**Fig. 1:**
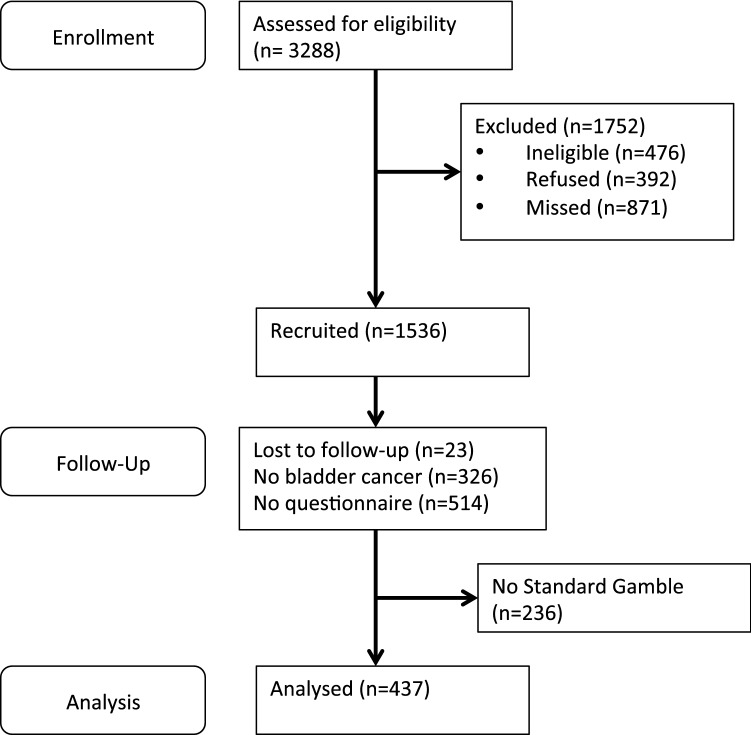
Flow chart depicting patient recruitment in the Bladder Cancer Prognosis Programme (BCPP) cohort study and inclusion for the current study

During the recruitment period (2005–2011), 1536 participants were enrolled. Of these 1536 patients, 326 were excluded because they did not have primary bladder cancer, another 23 were lost to follow-up, and 514 were excluded as they did not complete the first questionnaire, leaving 673 patients potentially available for assessment see Fig. 1. Of these 673 patients, 437 (65%) responded to the standard gamble section and were included in the analyses. These 437 participants were slightly different to the 236 who did not complete the standard gamble with regards to gender (*p* = 0.05) and age (*p* = 0.04), whereby the non-responders were older and more likely to be female (25% in non-responders vs. 19% in responders, *p* = 0.05).

Of the 437 study participants, 354 were male and 83 were female, the mean age was 69 years old, with a minimum of 33 and maximum of 90 years. 309 patients (73%) had a partner with whom they were married or living together. The median overall HRQoL as measured by the QLQ-C30 was 75 interquartile range (IQR: 58—83), and the median social support was 100 (IQR: 88—100) Table 1.

**Table 1:** Patient characteristics of all patients included in the analysis

Age			
Mean (SD)	68.8	(10.4)	
Range	33–90		
Sex			
Male (%)	348	(81%)	
Female (%)	82	(19%)	
Marital status			
With partner (%)	305	(73%)	
Without partner (%)	111	(27%)	
General health			
Median (IQR)	75	(58.3–83.3)	
Range	8–100		
Social support			
Median (IQR)	100	(87.5–100)	
Range	0–100		
Stage			
NMIBC			
pTis (%)	6	(1%)	
pTa (%)	245	(57%)	
pT1 (%)	238	(32%)	
MIBC			
pT2 (%)	37	(9%)	

Non-muscle-invasive bladder cancer (NMIBC)

Muscle-invasive Bladder Cancer (MIBC)

187 patients (43%) would not change from a cystoscopy to a biomarker unless the biomarker had a sensitivity of 100%. In fact, the median value of MAS was 99.9999%, and nearly 85% of patients indicated that they would require a sensitivity of at least 99% before preferring a urinary biomarker test over cystoscopy.

We did not observe any statistically significant associations between the MAS and age (*p* = 0.092), HRQoL (*p* = 0.161), or social support score (*p* = 0.566) Table 2. The distribution of MAS scores across the categorical variables of gender (*p* = 0.127), marital status (*p* = 0.374) and level of educational attainment (*p* = 0.060) did not differ, and no effect modification was found Table 3.

**Table 2:** Results from Spearman rank correlation analysis between MAS, age, general health and social support score

Age	
Spearman's rho	− 0.081
*p* value	0.092
General health	
Spearman's rho	0.087
*p* value	0.161
Social support score	
Spearman's rho	0.034
*p* value	0.566

**Table 3:** Results from Mann–Whitney *U* and Kruskal–Wallis test comparing rank sums of MAS between sex, marital status and NVQ levels

Variable	*n* per category	Rank sum
Sex		
Male	348	76,010
Female	82	19,693
* p* value of Mann–Whitney *U* test		0.127
Marital status		
With partner (%)	305	66,297
Without partner (%)	111	22,957
* p* value of Mann–Whitney *U* test		0.374
National Vocational Qualifications (NVQ)		
Level 1	43	5124
Level 2	57	7896
Level 3	81	10,513
Level 4	33	4513
Level 5	37	3579
*p* value of Kruskal–Wallis test		0.060

## Discussion

Our results indicate that the large majority of patients recently diagnosed with bladder cancer demand a very high level of sensitivity before they would be willing to accept urinary biomarkers as an alternative to cystoscopy for periodic surveillance. Based on the conclusions of Vriesema et al. (2000) [20], we expected that our population of cystoscopy naïve patients would indicate a lower MAS and therefore have a larger spread of responses. However, we observed the opposite. When comparing the results of previous studies to our results, it is clear that patients choose test certainty over test burden.

In all three studies [20, 21], more than 65% of the patients indicated that they would require an MAS of over 99% and approximately 90% of patients would require an MAS of over 90% Table 4. None of the existing biomarkers are able to consistently achieve this level of sensitivity.

**Table 4:** MAS distribution in the three different studies

MAS group	< 90	90 to < 99	99–100	Total
Vriesema et al.	9 (10.6)	18 (21.2)	58 (68.2)	85
Yossepowitch et al.	7 (3.5)	53 (26.5)	140 (70.0)	200
Nekeman et al.	27 (6.3)	38 (8.8)	365 (84.9)	430
Total	43 (6)	109 (15)	563 (78)	715

The high percentage of patients that would not prefer a MAS with sensitivity below 100% may reflect less than a full understanding of the concept of sensitivity, and also the fact that these patients were presented with a hypothetical situation. However, based on qualitative feedback from the research nurses who administered the standard gamble, it is our impression that many patients were “terrified” of the possibility of missing a tumour.

It is important to place these findings in the context of conventional white light cystoscopy as the “gold standard” for the diagnosis of NMIBC. However, new optical technologies such as photodynamic diagnosis and narrow band imaging [27] have shown that the sensitivity of conventional white light cystoscopy itself is much less than 100%, and that recurrent tumours are missed in up to 41% of patients [28]. Most patients in this study will have been unaware of the shortcomings of conventional white light cystoscopy and thus believed and readily accepted the 100% sensitivity that we used as a cystoscopy benchmark against which biomarkers were to be compared.

We also hypothesised that patients who perceive the burden of the cystoscopy to be high would be more inclined to choose a lower MAS. In a similar manner, patients with a low health-related quality of life (HRQOL) would find the burden of the cystoscopy to be higher and thus choose a lower MAS. In addition, we hypothesised patients with more social support would be willing to take more risk and thus have a lower MAS. Education was taken into account as we hypothesised that patients with a higher education would better understand the risk associated with cystoscopy and the benefit of having a urinary biomarker, even if it comes at the cost of sensitivity. We demonstrated that there was no significant association between MAS and National Vocational Qualifications level, although the highest levels (level four & five) seemed to report lower MAS values more often (*p* = 0.060).

Even though there was a moderate to high response rate (65%) in a patient population, we found little evidence of any significant difference between the patients who did and did not take part in the survey. Nevertheless, we cannot rule out the possibility that the MAS of the larger population might be somewhat different from that of our study sample. Furthermore, with bladder-preservation strategies being used more frequently for the treatment of muscle-invasive bladder cancer MIBC) with curative intent [29], one should recognise that this group of patients also require long-term cystoscopic surveillance; their opinions and MAS may differ from those of NMIBC patients. With only a relatively small sample of MIBC patients in this study (*n* = 37, 9%), we have been unable to make this comparison adjusting for any patient characteristics, although it should be noted that the lowest reported MAS was 90% in MIBC patients, while 10.5% (*n* = 43) of NMIBC patients reported a MAS of 90% or lower.

Two previous studies by Vriesema et al. and Yossepowitch et al. reported incongruent findings of associations between age and MAS, and gender and MAS; neither study was able to replicate the findings of the other [20, 21]. Our study did not replicate findings of either of the two previous studies. This lack of association across the studies between the various potential risk factors and MAS might be due to the small variance in MAS. Additionally, it might be possible that we did not find an association between age and MAS, because mostly older patients did not complete the questionnaire.

Moreover, patients were newly diagnosed when they filled in the questionnaire, increasing the observer-expectancy effect as the subject is in a temporary, vulnerable mind state and the research nurse might show empathy with the patient while a neutral stance is expected. Also, patients were presented with cystoscopy as a 100% accurate test as their reference, while the actual accuracy of cystoscopy is lower, especially in diagnosing low-grade disease. Future studies should consider rephrasing questions about MAS to make clear that the reference test is not by definition 100% accurate. Both caveats could increase the risk of inappropriately interpreting results.

Our results suggest that the bladder cancer biomarker research and development community, both commercial and academic, needs to aim for test sensitivity equal to or better than cystoscopy before such tests can be implemented into NMIBC surveillance regimens. This is unlikely to be achievable with the current generation of urinary biomarkers but may be achievable in the near future with urinary DNA-based approaches [16]. Future research should also focus on costs of such non-invasive biomarkers, for example, by investigating how much patients are willing to pay for each increment in diagnostic accuracy. Cystoscopy is unlikely to be replaced today, but tomorrow’s urinary biomarkers may allow the frequency of cystoscopic surveillance to be reduced.

In conclusion, our study results indicate that patients demand more sensitive urinary biomarkers than are currently available, thus patients choose certainty over burden.
